# Unveiling the Decarboxylation of a Malonic Acid‐Containing Macrocycle at Acidic pH: Implications and Applications of an Overlooked Phenomenon

**DOI:** 10.1002/cplu.202500213

**Published:** 2025-06-10

**Authors:** Sara Franchi, Marianna Tosato, Valerio Di Marco

**Affiliations:** ^1^ Department of Chemical Sciences University of Padova 35131 Padova Italy; ^2^ Radiopharmaceutical Chemistry Laboratory Nuclear Medicine Unit AUSL‐IRCCS Reggio Emilia 42123 Reggio Emilia Italy

**Keywords:** acidity constants, complexation, decarboxylation, Kryptofix 22, malonic acid, speciation

## Abstract

1,10‐Diaza‐18‐crown‐6‐1,10‐bis(malonic acid) (oddm or macromal) is a promising chelator for large hard metal cations due to its unique structure, which combines the complexing properties of aza‐crown Kryptofix 22 with those of malonic acid. This study uncovers the previously overlooked decarboxylation of macromal under mildly to strongly acidic pH conditions at room temperature. As a result, the acidity constants of macromal are carefully re‐evaluated, providing precise data for its accurate and informed handling in aqueous media. The implications of these findings for metal complexation and new possible applications of macromal are also presented.

## Graphical abstract

A malonic acid‐containing macrocycle is observed to spontaneously undergo decarboxylation in water at mildly to strongly acidic pH and at room temperature. This phenomenon has significant implications for its use in aqueous solution within various areas of chemistry, like complexation of metals.

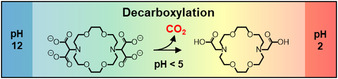

## 1 Introduction

1,10‐Diaza‐18‐crown‐6 (Kryptofix 22) and its derivatives are of significant interest due to their ability to complex large hard metal ions, including heavy alkaline, alkaline earth, and *f*‐block cations.^[^ ^1^, ^2^, ^3^, ^4^, ^5^ ^]^ The secondary amines of Kryptofix 22 can be easily functionalized by nucleophilic substitution, making this macrocyclic scaffold versatile for numerous applications, spanning from solubilization of heavy metals^[^ ^6^, ^7^ ^]^ and electrochemical sensors^[^ ^8^, ^9^ ^]^ to medicinal chemistry.^[^ ^5^, ^10^ ^]^ Functionalizing chelators with carboxylic acids is a very common strategy to enhance metal binding, as these highly acidic groups provide strong interactions with hard metals and can be easily handled during synthetic procedures.^[^ ^11^ ^]^

The combination of the above‐mentioned features makes 1,10‐diaza‐18‐crown‐6‐1,10‐bis(malonic acid) a highly promising chelator for large hard and borderline metals. First introduced in the ‘80s under the name “oddm”, this chelator demonstrated the ability to effectively complex Ba^2+^, Sr^2+^, Ca^2+^, Pb^2+^, Cd^2+^, and Eu^2+^ but was later abandoned.^[^ ^6^, ^12^, ^13^, ^14^ ^]^ Due to its potential, we were interested in reviving the chemistry of oddm, possibly opening the way to new applications, especially in aqueous solutions. To better reflect its structure, oddm is herein renamed “macromal”, emphasizing the presence of a macrocyclic scaffold and malonic acid moieties in this compound.

Remarkably, we discovered that macromal is susceptible to a degradative process at room temperature and mildly acidic conditions (pH < 5), due to a peculiar tendency to undergo decarboxylation. However, this degradation was overlooked by previous studies,^[^ ^6^, ^12^, ^13^, ^14^ ^]^ which assumed that macromal would decarboxylate only at extremely acidic pH (<2).^[^ ^13^ ^]^ This hypothesis was based on the properties of *N*‐*N*’‐ethylenebis(aminomalonic) acid (EAMA), which had been observed to be stable against decarboxylation at pH > 2.5–3 by Mashihara et al.^[^ ^15^ ^]^ As a consequence, the characterization of macromal reported in the literature, including, for instance, acidity constants,^[^ ^12^, ^13^, ^14^ ^]^ has to be considered with caution. This work provides strong experimental evidence for this degradation pathway and carefully re‐determines the acidity constants (p*K*_a_) of macromal.

## 2 Results and Discussion

**Scheme 1:**
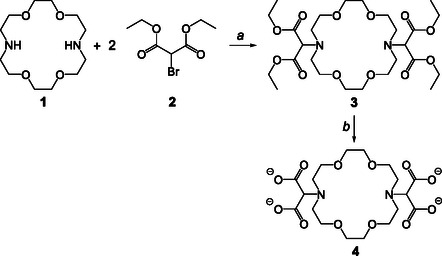
Synthesis of macromal (**4**) in its basic (deprotonated) form: a) Et_3_N, anhydrous dimethylformamide, N_2_, *T* = 80 °C, overnight and b) 1 M NaOH, H_2_O, *T* = 100 °C, 3 d.

**Figure 1:**
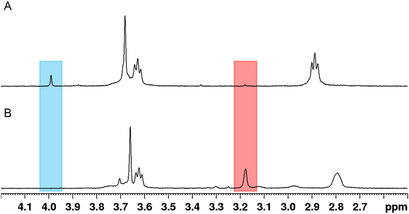
^1^H NMR spectra (90% H_2_O + 10% D_2_O, 400 MHz, *T* = 25 °C, *C*_macromal_ = 10^−3^ M, *I* = 0.15 M NaCl) of solutions obtained A) by dissolving macromal at autogenous pH (pH 10) and B) by dissolving macromal in aqueous HCl at pH 2 before setting the pH to 10. The blue and red rectangles show the two main signals that distinguish the two spectra (see text).

Macromal (**4**) was synthesized by employing a novel two‐step procedure by alkylation of Kryptofix 22 (**1**) with diethyl bromomalonate (**2**) through an S_N_2 reaction, followed by deprotection with NaOH, as illustrated in **Scheme** **1** and detailed in the Supporting Information. Compound **4** was obtained in its basic (deprotonated) form as a sodium salt, and the corresponding ^1^H NMR spectrum in aqueous solution is shown in **Figure** **1**A.

Unexpectedly, a significantly different ^1^H NMR spectrum was obtained when macromal was first dissolved in aqueous HCl at pH 2 and then brought to the same alkaline pH (Figure 1B). A comparison of the two spectra (Figure 1B vs. A) mainly pointed out the disappearance of the singlet at 3.99 ppm (blue rectangle) together with the appearance of another singlet at 3.18 ppm (red rectangle). These results suggested that a chemical transformation occurred, highlighting the need for a more detailed and thorough investigation. Therefore, two different sets of measurements were compared: increasing pH (from 2 to 12) and decreasing pH (from 12 to 2) titrations, both followed by either ^1^H NMR spectroscopy or pH‐potentiometry.

**Figure 2:**
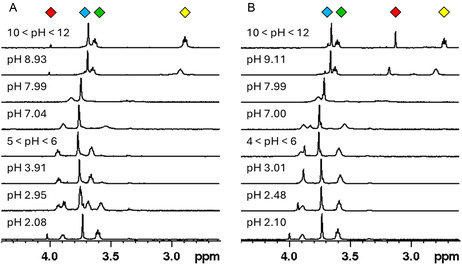
Comparison of ^1^H NMR titrations (90% H_2_O + 10% D_2_O, 400 MHz, *T* = 25 °C, *C*_macromal_ = 10^−3^ M, *I* = 0.15 M NaCl) of solutions initially containing macromal carried out with two opposite methods: A) by decreasing pH from 12 to 2 with HCl and B) by increasing pH from 2 to 12 with NaOH. Colored diamonds represent the attribution of signals to the corresponding ^1^H nuclei (see Figure 5).

**Figure 3:**
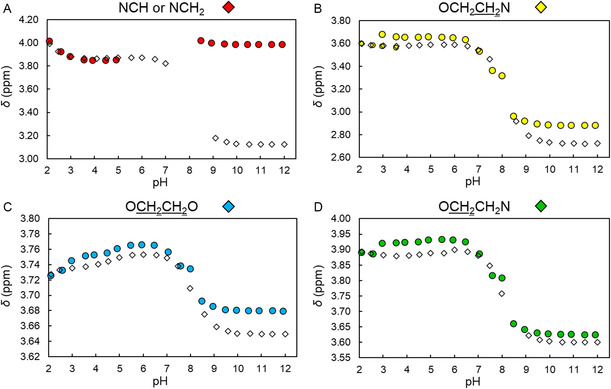
Comparison of the trends of chemical shift versus pH for each signal A,B,C,D) in the ^1^H NMR titrations of solutions initially containing macromal (90% H_2_O + 10% D_2_O, 400 MHz, *T* = 25 °C, *C*_macromal_ = 10^−3^ M, *I* = 0.15 M NaCl). Data were taken from the spectra depicted in Figure 2 and are relative to the titrations carried out with the two opposite procedures, i.e., by decreasing pH from 12 to 2 with HCl (colored circles) and by increasing pH from 2 to 12 with NaOH (white diamonds). B) In the pH range 3–4, two different signals attributed to OCH_2_ CH_2_N protons were discernible during the decreasing pH titration. Colors represent the attribution of signals to the corresponding ^1^H nuclei, as shown in Figure 5.

Representative ^1^H NMR spectra of macromal at different pH values for the two procedures are depicted in **Figure** **2**. The differences between the two sets of spectra are emphasized in **Figure** **3**, where the trend of chemical shift versus pH is reported for each signal.

**Figure 4:**
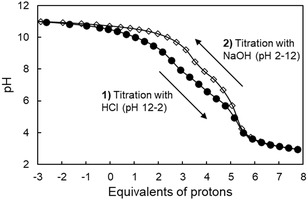
Comparison of two consecutive potentiometric titrations of the same solution initially containing macromal (*T* = 25 °C, *C*_macromal_ = 10^−3^ M, *I* = 0.15 M NaCl), **1)** first by decreasing pH from 12 to 2 with HCl (black circles) and **2)** afterwards by increasing pH from 2 to 12 with NaOH (white diamonds). Abscissa axis reports the equivalents of proton content relative to macromal, calculated as the difference between added H^+^ and OH^−^ moles normalized by macromal moles $(n_{H^{+}}-n_{{\text{OH}}^{‐}}\text{)/}n_{\text{macromal}}$.

A striking disagreement emerged also from pH‐potentiometric titrations performed with the two opposite methods. Not only the normalized titration curves (**Figure** **4**) but also the number and values of the resulting p*K*_a_ (**Table** **1**) were discordant. These experiments pointed out that macromal underwent an irreversible chemical modification at acidic pH, yielding a different compound, which accounted for the contradictory ^1^H NMR spectra and potentiometric titration curves when the pH was scanned from 2 to 12, compared to scans performed from 12 to 2.

**Table 1:** Comparison of the p*K*_a_ values obtained in this work from decreasing and increasing pH potentiometric titrations of macromal. Literature values for (supposed) macromal (oddm) and the bis‐acetic acid analogue odda (or dacda) are also listed for comparison.

	p*K*_a1_	p*K*_a2_	p*K*_a3_	p*K*_a4_
This worka) (decreasing‐pH)	6.4 ± 0.1	6.6 ± 0.2	8.5 ± 0.1	9.21 ± 0.08
This worka) (increasing‐pH)	3.99 ± 0.06	7.68 ± 0.08	8.2 ± 0.1	–
Literature for (supposed) macromal (oddm)	3.03b)	7.35b)	7.95b)	–
	3.06c)	7.44c)	7.55c)	–
	4.02d)	7.95d)	8.62d)	–
	3.31e)	8.00e)	8.60e)	–
Literature for odda (or dacda)	2.00f)	7.89f)	8.02f)	–
	2.90g)	7.80g)	8.45g)	–
	2.50h)	8.01h)	8.93h)	–
	2.24e)	7.99e)	8.95e)	–

a) *T* = 25 °C, *I* = 0.15 M NaCl.

b) *T* = 25 °C, *I* = 0.15 M NaCl, ref. [12].

c) *T* = 25 °C, *I* = 0.15 M KNO_3_, ref. [13].

d) *T* = 25 °C, *I* = 0.15 M NMe_4_Cl, ref. [13].

e) *T* = 25 °C, *I* = 0.1 M NMe_4_Cl, ref. [14].

f) *T* = 25 °C, *I* = 0.1 M KNO_3_, ref. [17].

g) *T* = 25 °C, *I* = 0.1 M NMe_4_Cl, ref. [2].

h) *T* = 25 °C, *I* = 0.1 M NMe_4_NO_3_, ref. [3].

We hypothesized that macromal could undergo decarboxylation, i.e., release one or two CO_2_ molecules, at acidic pH. This phenomenon is well known to occur in carboxylic acids bearing a carbonyl or another carboxylic group at position 3, but only under heating and/or in a strongly acidic medium.^[^ ^16^ ^]^ As stated in the Introduction, this was the reason why decarboxylation had been ruled out for macromal at pH > 2 and room temperature in previous studies.^[^ ^12^, ^13^, ^14^ ^]^

**Figure 5:**
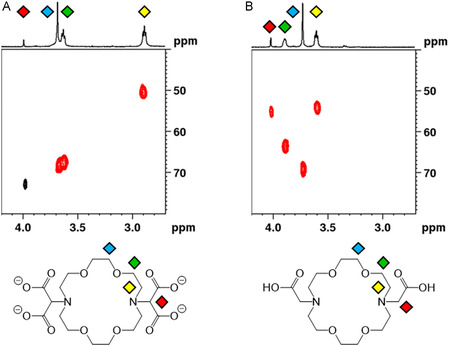
^1^H–^13^C multiplicity‐edited HSQC spectra (90% H_2_O + 10% D_2_O, 400 MHz, *T* = 25 °C, *C*_macromal_ = 10^−3^ M, *I* = 0.15 M NaCl) of a solution initially containing macromal at pH 12 and brought to A) pH 9.93 and B) pH 2.08. Positive (black) signals originate from CH or CH_3_ groups, and negative (red) signals originate from CH_2_ signals. Colored diamonds represent the attribution of signals to the corresponding ^1^H/^13^C nuclei.

We checked the occurrence of decarboxylation of macromal by performing multiplicity‐edited heteronuclear single‐quantum coherence (HSQC) spectroscopy. The spectrum of macromal dissolved at basic pH revealed the expected signals (i.e., negative CH_2_ for the ring and positive CH for the side moieties), substantiating the existence of macromal and its stability under these conditions (**Figure** **5**A). However, the signal characteristic of the side chains became negative when the solution was acidified to pH 2, revealing the transformation of CH into CH_2_ and providing strong evidence of decarboxylation (Figure 5B). Moreover, the low number of signals and cleanliness of the spectra suggest a high degree of symmetry of the molecule at both acidic and basic pH. This observation implies that both malonic groups undergo decarboxylation at low pH, converting the original bis‐malonic derivative (macromal) into the bis‐acetic analogue, which is named “odda” (or “dacda”) in the literature.^[^ ^2^, ^3^, ^14^, ^17^ ^]^

This phenomenon is detectable in the decreasing‐pH ^1^H NMR spectra starting at around pH 5, where macromal is still predominant and only traces of degradation products are visible (Figure 2A). The decarboxylation is slow and/or not complete in the pH range 4–5, while it becomes complete and almost immediate at pH 2–3 (Figure 2A).

**Scheme 2:**
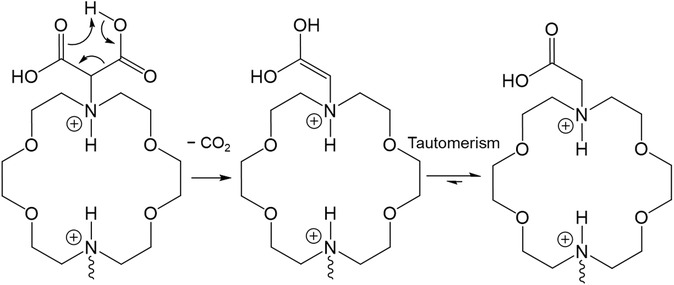
Proposed mechanism for the decarboxylation of macromal at acidic pH.

A possible explanation of the easiness by which macromal undergoes decarboxylation, compared to malonic acid, is the presence of two electron‐withdrawing groups at position 3 relative to each carboxylic group, i.e., the adjacent carboxyl and the tertiary amine that is protonated and bears a positive charge at acidic pH (**Scheme** **2**). The relatively high basicity of amines in macromal would therefore also explain why this compound undergoes decarboxylation at a higher pH than that reported for EAMA.^[^ ^15^ ^]^

After confirming the decarboxylation of macromal, its acid–base properties were explored as a crucial step preceding any application of this compound in aqueous solution. For instance, in metal complexation, the metal–chelator affinity is strongly influenced by the acid–base behavior of the compound. Protons compete with metal ions for binding at donor sites that display acid–base characteristics, highlighting the importance of understanding the solution properties of the chelator.^[^ ^4^, ^18^, ^19^ ^]^

The acidity constants (p*K*_a_) of macromal were published by two research groups who performed increasing‐pH potentiometric experiments starting from acidic pH.^[^ ^12^, ^13^, ^14^ ^]^ However, we observed that the previously reported p*K*_a_ for (supposed) macromal closely resemble those of odda, similarly to the values optimized in this work through potentiometry in the pH range from 2 to 12 (Table 1). This suggests that odda was present in the increasing‐pH titrations, thereby altering the p*K*_a_ values determined in these measurements. In view of the previous considerations, the determination of reliable p*K*_a_ values for macromal necessitates decreasing‐pH titrations starting at basic pH and stopping at pH ≈ 5 to ensure the integrity of the analyte.

The first row in Table 1 reports the p*K*_a_ obtained in this work by decreasing‐pH potentiometric titrations supported by ^1^H NMR, based on the data exemplified in Figure 2A and 4. The two highest p*K*_a_ (8.5 and 9.21) are attributed to the deprotonation of tertiary ammonium groups in the macrocyclic ring, while the other two (6.4 and 6.6) are relative to the deprotonation of carboxylic acids. Deprotonation of the other two carboxylic groups was not detected under the adopted conditions: the corresponding p*K*_a_ are expected to be very acidic, making their determination hampered by the instability of macromal at low pH.

## 3 Conclusion

This work revisited the aqueous chemistry of macromal, demonstrating that its malonic acid moieties are prone to decarboxylation at mildly to strongly acidic pH and at room temperature. This discovery is crucial and must be considered before any use of macromal in aqueous media, as its instability can lead to incorrect assessments of its properties, including not only acidity constants as demonstrated herein but also its chelation capacity. For instance, the stability constants for metal–macromal complexes reported by previous authors^[^ ^12^, ^13^, ^14^ ^]^ should be considered with caution. In fact, measurements starting at acidic pH can lead to solutions containing either macromal, odda, or a mixture of both chelators with variable compositions. The latter strongly depend on the experimental pH values and the waiting times after mixing the components in solution.

On the other hand, the easy decarboxylation of macromal opens new possibilities as well. For example, this compound might become of interest for applications requiring a controlled and localized release of CO_2_, easily triggered by mildly to highly acidic pH in an aqueous environment, spanning from gas‐assisted cancer therapy^[^ ^20^ ^]^ to wound healing.^[^ ^21^ ^]^

Furthermore, this research emphasizes the importance of supporting pH‐potentiometric data with qualitative information from a complementary technique sensitive to the molecular structure of the analyte (e.g., NMR). Integrating these approaches is especially critical when dealing with molecules containing potentially unstable functional groups, as is the case of macromal.

## Conflict of Interest

The authors declare no conflict of interest.

## Supporting information

Supplementary Material

## Data Availability

The data that support the findings of this study are available from the corresponding author upon reasonable request.
