# Supplementary material for: Unveiling the Decarboxylation of a Malonic Acid‐Containing Macrocycle at Acidic pH: Implications and Applications of an Overlooked Phenomenon
Source: Chempluschem. 2025 Jun 10;90(8):e202500213. doi: 10.1002/cplu.202500213 (PMC12352729; doi:10.1002/cplu.202500213)
Supplement: Supplementary file 1 — Supplementary Material [file CPLU-90-e202500213-s001.pdf]

Supporting Information

Experimental Section

General

Solvents and reagents were obtained from commercial suppliers (Sigma-Aldrich, Merck, and Fluka) and used as received. Ultrapure water (18.2 MΩ·cm) was collected through a Purelab Chorus (Veolia) system. Thin layer chromatography (TLC) was performed on silica gel-coated aluminum plates 60 F_{254} (Merck), and silica gel (high-purity grade, 60 Å, 230-400 mesh, 40-63 μm, Merck) was used as the stationary phase for flash column chromatography.

Nuclear magnetic resonance (NMR) spectra were acquired with either a Bruker Avance III 500 (500 MHz) or a Bruker AMX 400 (400 MHz). Chemical shift (*δ*) is reported in parts per million (ppm). Spectra were calibrated with respect to the residual solvent peak for deuterated solvents, and with respect to 3-(trimethylsilyl)propionic acid sodium salt (TSP, *δ* = 0 ppm) for H_2O/D_2O mixed solvent.

Synthesis of macromal

Kryptofix 22 (**1**) (488 mg, 1.86 mmol, 1 eq), triethylamine (Et_3N, 520 μL, 3.72 mmol, 2 eq), and diethyl bromomalonate (**2**) (635 μL, 3.72 mmol, 2 eq) were dissolved in anhydrous DMF (20 mL) under N_2 atmosphere and the resulting mixture was heated at T = 80°C overnight. Ice-cold H_2O (150 mL) was added, and the mixture was extracted with ethyl acetate (3 x 150 mL). The organic fractions were combined, washed with fresh ice-cold H_2O (350 mL), dried over $MgSO_4$, and evaporated. The crude was purified *via* flash column chromatography (silica gel, DCM/MeOH from 95:5 to 70:30) to obtain **3** as a yellow oil (58.3 mg, 0.10 mmol, 5% yield). ^1H NMR ($CDCl_3$, 500 MHz, T = 25°C): 1.26 (t, J = 7.1 Hz, 12 H, CH_3), 2.99 (t, J = 5.3 Hz, 8 H, $OCH_2\underline{CH_2}N$), 3.58 (t, J = 5.7 Hz, 8 H, $O\underline{CH_2}CH_2N$), 3.59 (s, 8 H, $O\underline{CH_2CH_2}O$), 4.16-4.23 (m, 10 H, OCH_2 and NCH). $^{13}C\{^1H\}$ NMR ($CDCl_3$, 126 MHz, T = 25°C): 14.2 (CH_3), 53.1 ($OCH_2\underline{CH_2}N$), 61.5 (OCH_2), 69.3 (NCH), 70.7 ($O\underline{CH_2CH_2}O$), 71.2 ($O\underline{CH_2}CH_2N$), 168.6 (quaternary C). ESI-MS: m/Z = 579.0 $[M+H]^+$, 601.0 $[M+Na]^+$, 617.0 $[M+K]^+$.

To **3** (55.4 mg, 0.096 mmol, 1 eq), 1 M NaOH (672 μL, 0.67 mmol, 7 eq) and H_2O (3.5 mL) were added, and the solution was heated at T = 100°C for 3 d. Macromal sodium salt (**4**) (61.1 mg) was

quantitatively obtained as a white solid. ^{1}H NMR (D_2O, 500 MHz, T = 25°C): 2.89 (t, J = 5.1 Hz, 8 H, $OCH_2\underline{CH_2}N$), 3.63 (t, J = 5.2 Hz, 8 H, $O\underline{CH_2}CH_2N$), 3.68 (s, 8 H, $O\underline{CH_2CH_2}O$), 3.96 (s, 2 H, NCH). $^{13}C\{^{1}H\}$ NMR (D_2O, 126 MHz, T = 25°C): 51.2 ($OCH_2\underline{CH_2}N$), 68.2 ($O\underline{CH_2}CH_2N$), 68.8 ($O\underline{CH_2CH_2}O$), 73.4 (NCH), 177.0 (quaternary C) (**Figures S1-S3**). Elemental analysis (carbon and nitrogen) and ionic chromatography (sodium): C/N/Na mass ratio = calculated for $C_{18}H_{26}N_2Na_4O_{12} \cdot 3\ NaOH$ 7.7:1:5.7, found 7.9:1:5.6. *Note:* Several attempts (experimental parameters were changed, and two different MS instruments were used) to acquire the ESI-MS spectrum of (**4**) were not successful, likely due to fragmentation or degradation of macromal under these conditions.

Potentiometric Titrations

Solutions were prepared in ultrapure water with 0.15 M NaCl as ionic strength (I). Automatic acid-base potentiometric titrations of macromal ($C_{macromal}$ ~ 10^{-3} M) were carried out with Metrohm 715 Dosimat titrator and burets and a Metrohm 713 pH-meter. pH was measured using a Hamilton combined glass electrode (pH 0-14) previously calibrated by adding known amounts of HCl to water. The cell was thermostated at T = 25°C and CO_2 was removed by a N_2 flow during each experiment. A 0.1 M HCl solution was prepared by dilution from the concentrated one (Aldrich, 37%) and standardized against Na_2CO_3 (Aldrich, 99.95-100.5%), which had been previously heated at ~ 260°C for 1 h to remove H_2O and CO_2 traces. A CO_2-free 0.1 M NaOH solution was prepared by dissolving pre-washed pellets (Emsure, 99-100%), protected against carbonation, and standardized against the HCl solution. Potentiometric data were analyzed with PITMAP program. [22]

NMR Spectroscopy

Batch acid-base ^{1}H NMR titrations were performed at T = 25°C for solutions containing macromal ($C_{macromal}$ = 10^{-3} M, I = 0.15 M NaCl) and traces of TSP for calibration. The solvent was 90% H_2O + 10% D_2O and water signal was suppressed through the excitation sculpting pulse scheme. The pH was adjusted with small additions (~ µL) of HCl or CO_2-free NaOH solutions, and it was measured with a XS pH50 VioLab pH-meter (XS Instruments) and a Crison combined glass electrode (pH 0-14), previously calibrated with commercial buffers at pH 4.01 and 7.00 (XS Instruments). The waiting time between sample preparation and spectra acquisition was ~ 24 h at room temperature. ^{1}H-^{13}C multiplicity-edited heteronuclear single-quantum coherence (HSQC) spectra were also acquired.

This technique provides a ^{1}H-^{13}C HSQC spectrum in which multiplicity information is available like that of a ^{13}C distortionless enhancement by polarization transfer (DEPT) 135, *i.e.* CH and CH_3 appear as positive signals while CH_2 appear as negative signals.

Supporting Figures

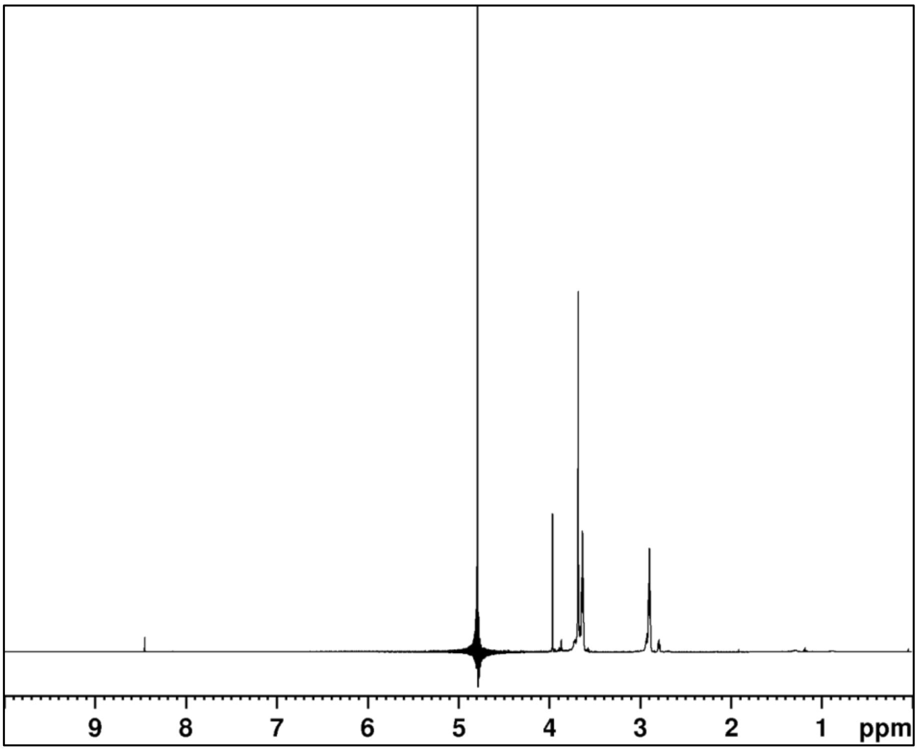

Figure S1. ^{1}H NMR spectrum of macromal (D_2O, 500 MHz, T = 25°C). 4.79 ppm = residual solvent peak; 8.45 ppm = traces of residual DMF; small peaks close to the main ones are likely due to different protonation states of the compound.

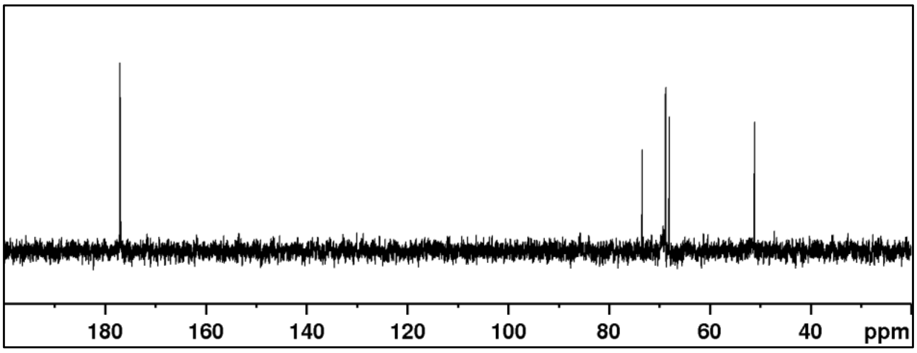

Figure S2. $^{13}C\{^{1}H\}$ NMR spectrum of macromal (D_2O, 126 MHz, T = 25°C).

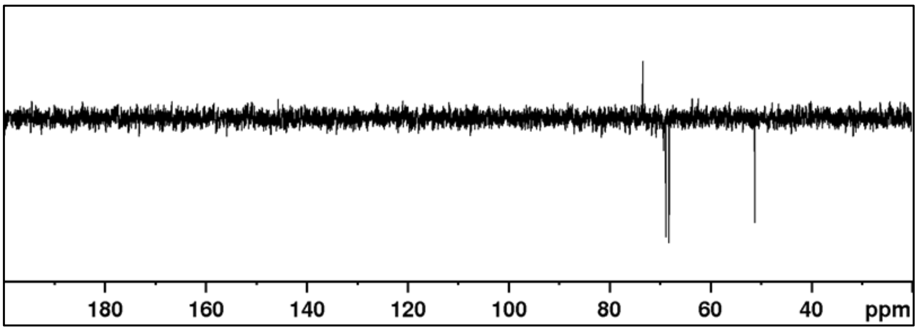

Figure S3. $^{13}C\{^{1}H\}$ DEPT-135 NMR spectrum of macromal (D_2O, 126 MHz, T = 25°C).
